# Occupational physicians’ practices in supporting employees with long COVID: a mixed-methods study

**DOI:** 10.1093/joccuh/uiaf078

**Published:** 2025-12-29

**Authors:** Yu Igarashi, Seiichiro Tateishi, Tomoko Sawajima, Arisa Harada, Juri Matsuoka, Mika Kawasumi, Koji Mori

**Affiliations:** Occupational Health Training Center, University of Occupational and Environmental Health, Japan, Fukuoka, Japan; Disaster Occupational Health Center, Institute of Industrial Ecological Sciences, University of Occupational and Environmental Health, Japan, Fukuoka, Japan; Tokyo Health Care Office, Health Care Center, Central Japan Railway Company, Tokyo, Japan; Department of Occupational Health Practice and Management, Institute of Industrial Ecological Sciences, University of Occupational and Environmental Health, Japan, Fukuoka, Japan; Department of Occupational Medicine, School of Medicine, University of Occupational and Environmental Health, Japan, Fukuoka, Japan; Department of Occupational Health Practice and Management, Institute of Industrial Ecological Sciences, University of Occupational and Environmental Health, Japan, Fukuoka, Japan; Global Headquarters, HOYA Corporation, Tokyo, Japan; Department of Occupational Health Practice and Management, Institute of Industrial Ecological Sciences, University of Occupational and Environmental Health, Japan, Fukuoka, Japan; Department of Occupational Health Practice and Management, Institute of Industrial Ecological Sciences, University of Occupational and Environmental Health, Japan, Fukuoka, Japan

**Keywords:** occupational health, occupational physicians, long COVIDmixed-methods study, workers

## Abstract

**Objectives:**

This study examined the support provided by occupational physicians (OPs) in Japan to employees with long COVID, a condition that significantly affected workforce health during the pandemic.

**Methods:**

An exploratory cross-sectional mixed-methods design was employed, consisting of qualitative interviews followed by a questionnaire survey targeting OPs certified by the Japan Society for Occupational Health. The interviews explored actual experiences of supporting workers with long COVID, and the findings were used to develop the questionnaire. The survey and interview findings were integrated to describe overall occupational health (OH) practices.

**Results:**

Twenty OPs reported 30 cases of employees with long COVID in the interviews. Based on these findings, a questionnaire survey was conducted, yielding 182 valid responses. The integrated results showed that OPs most frequently reported “Main OH responses” such as active listening, return-to-work assistance, and lifestyle guidance. Measures such as explaining workers’ compensation applications and preparing lists of outpatient clinics were less frequently reported. For “Advice for employers,” limitation of overtime, reduction of workload, and telework were commonly reported, whereas demotion and reassignment were rarely reported.

**Conclusions:**

This study clarified how OPs in Japan supported workers with long COVID through diverse, context-dependent practices. The identified main OH responses and advice for employers provide a framework for understanding current practices. Developing practical case examples, structured assessment tools, and workplace guidelines, together with further research grounded in real-world practice, will enhance OPs’ ability to provide appropriate support and strengthen preparedness for future health crises.

## Teaser

- Occupational health (OH) professionals are recognized as playing a crucial role in supporting workers with long COVID. However, few studies have detailed the specific nature and frequency of such support. No previous empirical research has clarified how occupational physicians (OPs) in Japan are responding to this issue in practice.

- This study provides the first empirical classification and frequency analysis of OPs’ support for workers with long COVID in Japan, using a robust mixed-methods design. It identifies both widely adopted practices, such as telework, workload reduction, and flexible work arrangements, as well as less frequent practices, such as collaboration with treating physicians and, in some cases, support related to workers’ compensation. By integrating qualitative and quantitative data, the study offers a comprehensive picture of current OH practice.

- These findings provide an evidence base to inform the development of practical guidelines, structured assessment tools, and shareable case examples to support OPs’ work. They may contribute to strengthening OH responses to long COVID globally and improve preparedness for future public health crises involving emerging infectious diseases.

## 1. Introduction

Approximately 10%-20% of individuals who recover from COVID-19 subsequently develop long COVID,^1^ which has had a profound impact on the global workforce.^2^ The prevalence of long COVID is reportedly highest among the working-age population relative to other age cohorts.^3,4^ Long COVID results in diminished work productivity, increased absenteeism, and workforce attrition.^3^^-^^9^ In the United States alone, an estimated 1.8 million to 4.1 million workers have exited the labor market due to the effects of long COVID, representing a substantial loss equivalent to full-time employment.^10^ As of 2024, the cumulative number of COVID-19 cases in Japan was projected to exceed 70 million, indicating that a substantial portion of the workforce may be affected by long COVID.^11^ These findings underscore the urgent need for comprehensive occupational health (OH) support measures for workers affected by long COVID.

**Figure 1:**
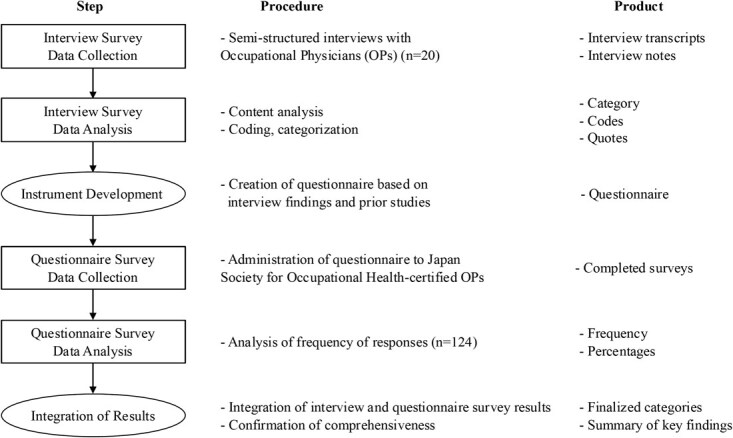
The sequential steps of the study, including interview data collection, qualitative analysis, instrument development, questionnaire administration, quantitative analysis, and results integration. For each step, the corresponding procedures and resulting study products are summarized, illustrating how interview and questionnaire data were systematically combined to generate the final study findings.

In response to the substantial and ongoing impact of long COVID on the working population and the corresponding need for effective OH support, numerous recommendations and guidance documents pertaining to return-to-work support for long COVID have been published.^12^^-^^22^ Various governmental bodies and professional organizations, including the American College of Occupational and Environmental Medicine, the European Agency for Safety and Health at Work, the Society of Occupational Medicine in the United Kingdom, the Ministry of Health, Labour and Welfare in Japan, and the Japanese Society of Occupational Health (JSOH), have issued guidelines specifically addressing long COVID, particularly with respect to facilitating return to work.^12^^-^^18^ Many studies have emphasized the central role of occupational physicians (OPs) in managing long COVID,^19^^-^^22^ highlighting the necessity of their involvement in workplace health protection and rehabilitation.

In Japan, the Industrial Safety and Health Act requires employers with 50 or more employees to appoint an OP to manage workplace health and provide “Advice for employers on OH” for workers with health issues.^23^ During the COVID-19 pandemic, OPs played a pivotal role in implementing workplace infection control measures.^24^ Accordingly, OPs remain critical in delivering workplace support for long COVID in Japan within the Japanese OH system.

Although several reports have reviewed the roles and activities of OH professionals during the COVID-19 pandemic in general,^25^^-^^28^ empirical surveys examining how OPs have specifically supported workers with long COVID in real-world practice remain scarce. In particular, the evidence base regarding OPs’ practical responses to long COVID has not yet been sufficiently established. Therefore, the aim of this study was to investigate the current status of support provided by OPs in Japan for workers with long COVID and to clarify the challenges they encounter in practice.

## 2. Methods

### 2.1. Research design

This study employed a cross-sectional mixed-methods framework.^29^ To address insufficient documentation of the practical activities of OH professionals and the limitations of existing methodological frameworks, we adopted an exploratory sequential design. First, we conducted qualitative interviews to capture the actual state of OH support for long COVID. Based on these findings, we developed a questionnaire survey to further assess and generalize the results.

In this study, we examined “Main OH responses to long COVID,” defined as specific practices and key insights derived from OPs’ experiences, and “Advice for employers on long COVID,” or recommendations given by OPs to employers regarding affected employees. These responses were systematically categorized to provide an overview of OH support for long COVID. To evaluate their implementation, we conducted a questionnaire survey to quantify their frequency and assess their generalizability. An open-ended section was also included to capture additional responses not covered by the pre-established categories.

The overall research design is illustrated in Figure 1. Of note, the term “long COVID” was not explicitly defined in either the questionnaire or interviews, as diagnostic criteria had not yet been fully established at the time of the survey.^30^

### 2.2. Interview survey

#### 2.2.1. Participants

Participants in the interview were OPs with experience in providing support to employees with long COVID, and were recruited broadly through snowball sampling. Invitations for participation were sent via email to 46 individuals, of whom 26 declined due to “insufficient experience.” Ultimately, informed consent was obtained from 20 participants. For OPs who had supported multiple employees, information was collected for each individual case, resulting in a total of 30 cases provided by the 20 participating OPs. Our target sample size was approximately 20 participants following the recommendations of Guest et al.^31^ As no additional novel insights emerged when 30 cases had been secured, indicating data saturation, further recruitment was considered unnecessary.

#### 2.2.2. Data collection

The interview survey was conducted online between October and December 2022 by video conferencing (Zoom; Zoom Video Communications, Inc, San Francisco, CA, USA). Semi-structured interviews were carried out based on a predeveloped interview guide. The main topics were: (1) an overview of the company; (2) individual workplace support experiences;(3) main OH responses to long COVID; and (4) advice for employers on long COVID. Each interview lasted approximately 60 to 90 minutes and was audio-recorded with the prior consent of participants. As an honorarium, each participant received by mail a gift certificate valued at 5000 yen. All recordings were transcribed verbatim by an external transcription service. No member checking of the transcripts was conducted.

#### 2.2.3. Data analysis

The verbatim transcripts and corresponding interview notes were summarized as key points by case and repeatedly reviewed by the research team.

#### Main OH responses to long COVID

Responses to the question, “What are the distinctive and key experiences of OPs in responding to long COVID?” were analyzed using Berelson’s content analysis methodology.^32^ Specifically, multiple team members initially identified excerpts (quotes) from the transcripts that reflected response behaviors or contextual factors unique to long COVID, and assigned a descriptive code to each quote to capture its essential meaning. For example:

The individual only thought about using paid leave, so I had the HR department confirm whether this qualifies as a work-related injury and asked them to provide the necessary information. (ID16)

Code: Explanation of workers’ compensation application.

Subsequently, codes with similar meanings were grouped and consolidated into broader categories. Final categorization was determined through consensus among the research team. The classification emphasized distinctions from other chronic conditions such as cancer, cardiovascular diseases, and rare diseases, and identified responses that were either distinctive for or key to long COVID.

#### Advice for employers on long COVID

For the segments pertaining to “Opinions on employment for employers” elicited during the semi-structured interviews, the research team conducted repeated reviews of the verbatim transcripts and accompanying interview notes. Specific recommendations of OPs were extracted as quotes, and corresponding codes were assigned to reflect their underlying meaning. Codes sharing similar meanings were then organized into broader categories with reference to previous studies and the practical expertise of the research team.^19,33^^-^^38^ The classification criteria emphasized whether the opinions were intended to prevent a deterioration in employee health due to long COVID or to mitigate risks related to workplace accidents and public hazards. These responses were broadly categorized as job accommodation measures.

The research team each possessed more than 8 years of professional experience as OPs and held specialist certification from JSOH. Additionally, all members had trained in qualitative research methods. This combined expertise and experience strengthened the methodological rigor and appropriateness of data collection and analysis.

### 2.3. Questionnaire survey

#### 2.3.1. Participants

The questionnaire survey was designed to assess the generalizability of OH practices in Japan and was distributed to 690 OPs and specialists certified by JSOH. Although this sample does not represent the entirety of OPs in Japan, it was considered an appropriate reference group.

#### 2.3.2. Development of the questionnaire

The questionnaire addressed respondent characteristics, the frequency of provision of individualized support, and the presence or absence of “Main OH responses to long COVID” and “Advice for employers on long COVID.” It also included open-ended sections for additional comments on the responses to ensure that no significant experiences fell outside the scope of the predefined items.

The “Main OH responses to long COVID” items were derived directly from the interview findings. The “Advice for employers on long COVID” component was expanded by incorporating additional items deemed relevant, based on interview results, the research team’s prior experience in employment support for long COVID and other conditions, as well as insights drawn from established guidelines and previous studies.^19,33^^-^^38^ This expansion was intended to ensure that potentially important types of advice not captured in the interviews were also included, thereby improving the comprehensiveness and validity of the questionnaire.

The final questionnaire consisted of 10 items on “Main OH responses to long COVID” derived from the interview survey, and 19 items on “Advice for employers on long COVID,” including 11 items from the interview findings plus 8 items informed by previous research. These additional items included assignment to work with longer deadlines, provision of flexible working hours, provision of staggered working hours, restrictions on domestic and international business trips, provision of time to attend medical appointments, modifications to the physical work environment, and permission not to wear a mask.

#### 2.3.3. Data collection

The questionnaire survey was conducted between April and June 2023. Invitations requesting participation were distributed by mail, and responses were collected anonymously via Google Forms. A single reminder notice was sent by mail during the survey period. No monetary or other incentives were provided to participants.

#### 2.3.4. Data analysis

The proportion of respondents who indicated having undertaken each specific support measure was calculated and analyzed descriptively. Frequently reported responses were interpreted as indicative of common trends in OH practice; however, the potential significance of responses was not diminished by infrequent reporting, as these may be meaningful in individual contexts. Accordingly, results were interpreted with consideration of both overall trends and the distinct significance of individual cases.

Responses in the open-ended sections were reviewed to evaluate the appropriateness and comprehensiveness of the existing classification framework, with particular attention to whether they could be accommodated within the predefined categories or indicated novel perspectives. Any responses that could not be classified under the existing framework were incorporated as newly identified support measures.

### 2.4. Integration of interview and questionnaire findings

This integration combined qualitative insights from interviews, which identified types of support, with quantitative data from the questionnaire, which assessed their frequency in practice, thereby providing a comprehensive and triangulated understanding of OPs’ support for long COVID.

### 2.5. Ethical considerations

This study was approved by the Ethics Review Committee of the University of Occupational and Environmental Health, Japan (Approval No. R4-042). Written informed consent was obtained from all interview participants, and the questionnaire survey was conducted anonymously, with completion regarded as consent. All data were anonymized to ensure confidentiality. The study was conducted in accordance with the Declaration of Helsinki and reported in line with the STROBE guidelines.

## 3. Results

### 3.1. Interview survey

A total of 30 cases of support provided to workers with long COVID were reported by 20 OPs. Thirteen participants (65.0%) were male; average length of experience as an OP was 11.3 years, and 18 participants (90.0%) were JSOH-certified. Of the respondents, 10 worked full-time as OPs in large companies, whereas the other 10 mainly served multiple small- and medium-sized enterprises through affiliations with OH organizations, universities, or independent offices. Geographically, respondents were distributed across multiple regions of Japan, including the Tokyo metropolitan area, Kinki, Tokai, Chugoku, Kyushu, and Tohoku.

#### Main OH responses to long COVID

The interview survey identified 10 categories of OH responses: (1) provision of return-to-work support, (2) active listening to employees’ concerns, (3) provision of lifestyle guidance, (4) explanation of workers’ compensation application, (5) explanation of the future outlook of long COVID, (6) explanation of long COVID to supervisors, (7) recommendation to visit outpatient clinics for long COVID, (8) compilation of a list of outpatient clinics capable of treating long COVID, (9) collaboration with the treating physician, and (10) recommendation of sick leave again. Illustrative examples of these responses are provided in Table 1.

**Table 1:** Categories of “Main OH responses to long COVID” identified from interviews with occupational physicians, with illustrative verbatim excerpts (Interviewee ID).

**No.**	**Category**	**Description**	**Example (Interviewee ID XX)**
**1**	Return-to-work support	Evaluation of readiness to return to work for employees with long COVID	*The employee expressed a desire to return to work, so we conducted a return-to-work interview. However, they were clearly not in a condition to resume work. They had made no real preparations for returning, so I provided advice on how to get ready for resumption.* (ID 1)
**2**	Active listening to employees’ concerns	Active listening to employees’ anxieties	*There were no particular attendance issues, so initially my main role was simply to listen when the employee came to consult.* (ID 14)
**3**	Provision of lifestyle guidance	Providing guidance on daily habits, such as sleep and exercise, to help alleviate symptoms	*The employee misunderstood and thought anaerobic exercise would be good, so I advised that if anything, light aerobic exercise within reasonable limits would be preferable, and emphasized not to push themselves to exhaustion.* (ID 10)
**4**	Explanation of worker compensation application	Explanation of workers’ compensation when long COVID may qualify for coverage	*The employee only thought about using paid leave, so I had the Human Resource staff confirm whether it would qualify as a work-related injury and asked them to provide the necessary guidance.* (ID 16)
**5**	Explanation of the future outlook of the long COVID	Providing explanations about the condition and the expected course of symptoms to employees	*I explained that it is serious because symptoms may develop in the future if it is long COVID.* (ID 15)
**6**	Explanation of long COVID to supervisors	Providing explanation of the condition and prognosis to supervisors	*There were some data on sequelae, so I made sure to explain it properly to the supervisor and to the employee as well, so they would have a realistic expectation of the course.* (ID 11)
**7**	Recommendation to visit outpatient clinics for long COVID	Recommendation to consult medical institutions capable of treating long COVID symptoms	*When they sought advice, I suggested visiting a doctor because I thought there was value in getting a proper diagnosis, arranging leave, and obtaining a medical certificate.* (ID 16)
**8**	Compilation of a list of outpatient clinics capable of treating long COVID	Compilation and provision of a list of outpatient clinics for long COVID to facilitate access	*A prefecture has a search website for long COVID clinics, so I checked the list and had the employee visit one. They later said the doctor confirmed it was indeed long COVID.* (ID 15)
**9**	Collaboration with the treating physician	Requesting medical information from the employee’s primary physician	*After hearing about the symptoms, I drafted a letter to the primary physician describing the difficulties the employee was experiencing.* (ID 9)
**10**	Recommendation of sick leave again	Recommendation of renewed leave due to severe symptoms	*The fatigue was so severe and their thinking so impaired that it was not something that could be managed with work restrictions or adjustments. I told them, “You should take leave,” and gave them a push in that direction. I suspect their usual self would have realized this immediately, but brain fog likely made it hard for them to judge.* (ID 9)

Abbreviation: OH, occupational health.

Categories were derived from qualitative content analysis of semi-structured interviews with occupational physicians. Each category represents a distinct type of occupational health response to support workers with long COVID. Examples are representative verbatim quotes that illustrate the meaning of each category.

#### Advice for employers on long COVID

The interview survey also extracted 11 categories of advice for employers: (1) telework, (2) limitation of overtime, (3) reduction of workload, (4) assignment to work with a high degree of discretion, (5) restriction of shift work or night shift, (6) reassignment, (7) demotion, (8) restriction of tasks worsening medical condition or safety risks, (9) restriction of tasks sensitive to reduced work capacity, (10) provision of a rest area or rest breaks, and (11) workplace environment adjustment. Illustrative examples of these recommendations are presented in Table 2.

**Table 2:** Categories of “Advice for employers on long COVID” identified from interviews with occupational physicians, with illustrative verbatim excerpts (Interviewee ID).

**No.**	**Category**	**Example (ID XX)**
**1**	Telework	*The employee had mild fever and fatigue that made commuting difficult, so after consulting with the employee and their department, we decided to trial remote work first. They began by working from home.* (ID 11)
**2**	Limitation of overtime	*Previously, the employee’s overtime was capped at about 20 hours per month, but when they returned to work this time, we significantly reduced the cap to within five hours per month.* (ID 8)
**3**	Reduction of workload	*They were handling complex assignments, so we discussed this with their supervisor and agreed to reduce their workload.* (ID 12)
**4**	Assignment to work with a high degree of discretion	*We arranged duties with reduced burden. Previously, they had to respond immediately if anything happened with their patients, so at first after returning, they started with more flexible, free-form tasks to help them adjust gradually.* (ID 16)
**5**	Restriction of shift work or night shift	*Since they still had insomnia, we removed them from shift work and arranged for daytime duties only, with no overtime allowed, so they could return while monitoring their condition.* (ID 5)
**6**	Reassignment	*They worked in an area where they might have needed to wear masks, so I submitted a written recommendation stating, “Tasks requiring dust or gas masks are unsuitable; please limit their work to desk duties for the time being.”* (ID 19)
**7**	Demotion	*When they returned to work, we negotiated with management to change their position and rank, moving them from an executive officer role down to a regular staff member. Naturally, their salary was reduced as well.* (ID 2)
**8**	Restriction of tasks worsening medical condition or safety risks	*It was during the peak heat stress season, so we suggested easing them back in by including more desk work to help them acclimate to the heat gradually.* (ID 8)
**9**	Restriction of tasks sensitive to reduced work capacity	*Because of decreased physical strength, we temporarily reassigned them within the security team to a relatively light-duty position, such as gate reception, so they could gradually build back up.* (ID 2)
**10**	Provision of a rest area/rest breaks	*We increased the proportion of back-office work and helped them adjust gradually. We also ensured they had a place in the back to rest whenever needed, without inconveniencing others.* (ID 2)
**11**	Workplace environment adjustment	*In the second consultation, they mentioned being overly sensitive to temperature, saying it felt extremely cold even in May or June. Although they tried to manage with blankets and extra layers, they still felt very cold, so we arranged for them to change seats.* (ID 20)

Categories were derived from qualitative content analysis of semi-structured interviews with occupational physicians. Each category represents a distinct type of advice provided to employers regarding support for workers with long COVID. Examples are representative verbatim quotes that illustrate the meaning of each category.

### 3.2. Questionnaire survey

The contents of the questionnaire are summarized in Table S1, and the results are described below.

#### 3.2.1. Respondent characteristics

A total of 184 participants responded to the questionnaire survey. Of these, 182 provided valid consent and were included in the final analysis, yielding a response rate of 26.4%. Among respondents, 114 (62.6%) were male, and the average duration of professional experience was 17.1 years. Detailed information such as company size or industry type was not collected.

#### 3.2.2. Individual workplace support experiences

Of the respondents, 124 (68.1%) had provided individualized support for workers affected by long COVID. The distribution of cases was as follows: 58 respondents (31.9%) reported no experience, 110 (60.4%) had handled 1-5 cases, 5 (2.7%) had supported 6-9 cases, and 9 (4.9%) had handled 10 or more cases; thus, approximately 70% of participating OPs had direct experience supporting employees with long COVID.

#### 3.2.3. Main OH responses to long COVID

Table 3 presents the frequency distribution for each category. The following items were most frequently reported: active listening to employees’ concerns (97.6%), provision of return-to-work support (86.3%), provision of lifestyle guidance (83.9%), recommendation to visit outpatient clinics for long COVID (82.3%), explanation of long COVID to supervisors (79.8%), and explanation of the future outlook of long COVID (71.8%). By contrast, measures such as explanation of workers’ compensation application (8.9%), compilation of a list of outpatient clinics capable of treating long COVID (13.7%), collaboration with the treating physician (29.8%), and recommendation of additional sick leave (34.7%) were less frequently cited among those with relevant support experience.

**Table 3:** Frequency of core OH response to long COVID workers identified through questionnaire survey (*n* = 124).

**Category of core OH response to long COVID**	***n***	**%**
**Active listening to employees’ concerns**	121	97.6
**Provision of return-to-work support**	107	86.3
**Provision of lifestyle guidance**	104	83.9
**Recommendation to visit an outpatient clinic for long COVID**	102	82.3
**Explanation of long COVID to supervisors**	99	79.8
**Explanation of the future outlook of long COVID**	89	71.8
**Recommendation of sick leave again**	43	34.7
**Collaboration with the treating physician**	37	29.8
**Compilation of a list of outpatient clinics capable of treating long COVID**	17	13.7
**Explanation of workers’ compensation application**	11	8.9

Abbreviation: OH, occupational health.

In the open-ended responses, 22 additional responses that were not consistent with the predefined items were noted, including support for employees’ families, explanations of residual symptoms to human resources personnel and colleagues, guidance on steroid inhalation, provision of detailed information to secondment destinations, referrals to medical institutions, instructions on visiting health care facilities, and recommendations for continued leave. These were incorporated into existing categories: “Explanation of the long-term outlook for long COVID,” “Explanation of long COVID to supervisors,” “Coordination with the treating physician,” and “Recommendation of sick leave again.” Guidance on steroid inhalation was incorporated into the category “Provision of lifestyle guidance.” No entirely new categories beyond those derived from the interview survey were identified.

#### 3.2.4. Advice for employers on long COVID

Table 4 presents the frequency distribution for each category. Fourteen respondents indicated that they had no experience providing “Advice for employers on long COVID.” Advice that was relatively common included limitation of overtime (63.7%), reduction of workload (50.8%), telework (44.4%), provision of time to attend medical appointments (20.2%), and restrictions on shift work and night shift (18.5%).

**Table 4:** Frequency of “Advice for employers on long COVID” identified through the questionnaire survey (*n* = 124).

**Category of “Advice for employers on long COVID”**	***n***	**%**
**Limitation of overtime**	79	63.7
**Reduction of workload**	63	50.8
**Telework**	55	44.4
**Provision of time to attend medical appointments**	25	20.2
**Restrictions on shift work and night shift**	23	18.5
**Restrictions on domestic and international business trips**	20	16.1
**Provision of flexible working hours**	17	13.7
**Provision of a rest area/rest breaks**	17	13.7
**Provision of staggered working hours**	16	12.9
**Restriction of tasks that may worsen medical conditions or pose safety risks**	16	12.9
**Permission to change commuting methods**	15	12.1
**Restriction of tasks sensitive to reduced work capacity**	12	9.7
**Reassignment**	7	5.6
**Assignment to work with a high degree of discretion**	6	4.8
**Adjustment of the workplace environment**	4	3.2
**Modifications to the physical work environment**	3	2.4
**Permission not to wear a mask**	2	1.6
**Assignment to work with longer deadlines**	1	0.8
**Demotion**	1	0.8
**No experience**	14	11.3

In the open-ended responses, 3 participants reported additional measures, including “maintaining distance from colleagues to mitigate the effects of persistent coughing,” “considering job reassignment in cases of prolonged health deterioration,” and “implementing flexible leave arrangements based on health status.” These responses were incorporated into the existing categories of “Adjustment of the physical workplace environment,” “Reassignment,” and “Provision of flexible working hours.” However, no new categories were identified beyond those already established for “Advice for employers on long COVID.”

### 3.3. Integration of interview and questionnaire survey results

#### 3.3.1. Integration of “Main OH responses to long COVID”

The interview survey identified 10 categories of “Main OH responses to long COVID.” These categories were subsequently included in the questionnaire survey. Integration of the findings showed that some responses, such as active listening to employees’ concerns, provision of return-to-work support, and provision of lifestyle guidance, were frequently reported in the questionnaire survey, whereas others, including explanation of workers’ compensation application and compilation of outpatient clinic lists, were less common.

#### 3.3.2. Integration of “Advice for employers on long COVID”

The interview survey generated 11 categories of “Advice for employers.” For the questionnaire survey, 8 additional items were incorporated based on prior studies and expert experience, resulting in 19 items in total. Integration of the findings showed that some measures, such as limitation of overtime, reduction of workload, and telework, were frequently reported in the questionnaire survey, whereas others, including demotion and reassignment, were rarely reported.

## 4. Discussion

This mixed-methods study clarified the current state of OP support for workers with long COVID in Japan. Two key findings emerged. First, OPs were more likely to provide direct, individualized support for workers, while measures that required institutional resources, external collaboration, or organizational adjustments were implemented less frequently. Second, advice for employers most often involved relatively simple workload modifications, whereas structural or more drastic measures were rarely reported. These findings indicate variability in OP responses, which likely reflects differences in case severity, workplace environments, and available resources rather than inconsistency or lack of knowledge among OPs. In some situations, collaboration with treating physicians or involvement in workers’ compensation procedures may simply not have been necessary. This variability, therefore, appears to represent appropriate contextual adaptation rather than a deficit in practice.

### 4.1. Individual workplace support experience

Approximately 30% of OPs reported no direct experience supporting workers with long COVID. This result should be interpreted with caution. It may reflect the relatively low incidence of severe cases requiring formal support, or variation across industries and company sizes in the appointment of OPs. Although further investigation is needed, this finding suggests that the scope of OP involvement may not have fully captured the total needs of workers affected by long COVID.

### 4.2. Main OH responses to long COVID

The main OH responses identified in the interviews and assessed in the questionnaire survey illustrate the primary ways in which OPs supported workers with long COVID. Frequently adopted practices included active listening, return-to-work support, and lifestyle guidance, all of which are considered appropriate for addressing common symptoms such as fatigue and uncertainty about recovery. In contrast, less frequently reported measures included collaboration with treating physicians, preparation of outpatient clinic lists, and, in some cases, providing explanations of workers’ compensation applications. These differences suggest that OPs were more likely to provide direct, individualized support. In contrast, measures involving coordination with external parties or organizational-level arrangements were reported less often.

### 4.3. Advice for employers on long COVID

Advice for employers on long COVID also showed variation in implementation frequency. Limitation of overtime, reduction of workload, and telework were the most frequently reported measures, consistent with previous findings on effective workplace accommodations. These measures are considered appropriate for addressing common long COVID symptoms, particularly fatigue and cognitive impairment. In contrast, structural measures such as demotion or reassignment were infrequently reported, which may reflect reluctance to implement substantial job changes. Telework was widely implemented during the pandemic, but many jobs remain unsuitable for remote work, highlighting the importance of alternative accommodations such as flexible scheduling, provision of rest areas, and adjustments to commuting arrangements. These findings indicate that OPs most often recommended accommodations that are feasible and adaptable across diverse workplace contexts and that correspond to common long COVID symptoms.

### 4.4. Implications

In the context of long COVID, some OP practices such as collaboration with treating physicians or support for workers’ compensation applications were reported less frequently, which may reflect systemic or procedural challenges in OH practice. These lower frequencies observed in the survey highlight structural barriers in OH systems, which were further underscored in the open-ended responses. Although workplace guidelines on infection prevention, business continuity planning, and needs assessments during pandemics have been developed by Tatemichi,^39^ these do not specifically address long COVID. Consequently, comprehensive and standardized guidance for occupational rehabilitation of workers with long COVID remains limited, and OPs are often required to make complex decisions under conditions of uncertainty. Moreover, evidence indicates that physical and mental exertion can exacerbate long COVID symptoms,^1,40^ and in this study approximately 30% of OPs reported recommending additional sick leave in persistent cases. This suggests that renewed leave was a relatively common response among OPs when symptoms continued. Based on this finding, clearer guidance on occupational rehabilitation and tools for assessing work capacity may help support decision-making in such cases. Finally, enhancing OPs’ ability to respond promptly to emerging health threats remains important. Continued development of professional education and practical resources may help support decision-making in real-world practice. Such efforts could contribute to improving current responses to long COVID and strengthening preparedness for future health crises.

### 4.5. Limitations

This study has several limitations. First, there is the possibility of recall bias, as participants were asked to report on past experiences related to COVID-19; however, because the survey was conducted during the pandemic, the magnitude of this bias is likely limited. Second, there is the potential for selection bias, as the sample consisted mainly of JSOH-certified experts and respondents working in larger companies. Therefore, the findings may not represent all OPs in Japan, particularly those in smaller enterprises where OH resources are often more limited. At the same time, the fact that the findings were drawn from a group engaged in OH activities at an advanced level can also be considered a strength. Third, the response rate was relatively modest (26.4%), with 182 valid respondents. Fourth, the survey was conducted in April 2023 and does not capture subsequent changes in infection status, awareness, or evolving responses to long COVID. Furthermore, OPs’ responses may vary by industry, employment conditions, and the design of workplace health management systems. Accordingly, further research is warranted to deepen understanding of OPs’ roles in addressing long COVID. Fifth, this study did not assess OPs’ knowledge, perceptions, or attitudes toward long COVID. Therefore, interpretations regarding the reasons underlying their practices should be made with caution.

## 5. Conclusions

This mixed-methods study clarified the current state of OP support for employees with long COVID in Japan. OPs commonly provided individualized measures such as active listening, return-to-work support, and workload adjustments, whereas practices that required coordination with external parties or organizational-level arrangements were less frequently implemented. OPs most commonly implemented individualized support measures, whereas practices requiring external coordination or organizational-level arrangements were reported less frequently. These findings illustrate variability in OP practices and indicate the need for practical tools, clear guidelines, and further research to strengthen OH responses to long COVID and enhance preparedness for future health crises.

## Supplementary Material

Web_Material_uiaf078

## Data Availability

The datasets generated during and/or analyzed during the current study are available from the corresponding author on reasonable request.
