# Supplementary material for: Occupational physicians’ practices in supporting employees with long COVID: a mixed-methods study
Source: J Occup Health. 2025 Dec 29;68(1):uiaf078. doi: 10.1093/joccuh/uiaf078 (PMC12965807; doi:10.1093/joccuh/uiaf078)
Supplement: Web_Material_uiaf078 [file web_material_uiaf078.docx]

**Supplementary Table 1. Questionnaire items on Main OH Responses and Advice for Employers on Long COVID”**

**1. Demographic Information**

Years of experience, Sex

**2. Experience with Individual Support for Workers with Long COVID**

Number of cases supported: 0 / 1–5 / 6–9 / 10 or more

**3. Please indicate whether you have experience providing support to workers with Long COVID. (Select all that apply) If applicable, please describe any other experiences in the space provided below. (Free Description)**

3.1. Provision of return-to-work support

3.2. Active listening to employees’ concerns

3.3. Provision of lifestyle guidance

3.4. Explanation of workers’ compensation application

3.5. Explanation of the future outlook of Long COVID

3.6. Explanation of Long COVID to supervisors

3.7. Recommendation to visit outpatient clinics for Long COVID

3.8. Compilation of a list of outpatient clinics capable of treating Long COVID

3.9. Collaboration with the treating physician

3.10. Recommendation of sick leave again

**4. Please indicate whether you have provided advice to employers regarding workers with Long COVID. (Select all that apply.) If applicable, please describe any additional experiences in the space provided below. (Free Description)**

4.1. Telework

4.2. Limitation of overtime

4.3. Reduction of workload

4.4. Assignment to work with a high degree of discretion

4.5. Assignment to work with longer deadlines

4.6. Provision of flexible working hours

4.7. Provision of staggered working hours

4.8. Restrictions on domestic and international business trips

4.9. Restrictions on shift work and night shift

4.10. Reassignment

4.11. Demotion

4.12. Restriction of tasks that may worsen medical conditions or pose safety risks

4.13. Restriction of tasks sensitive to reduced work capacity

4.14. Permission to change commuting methods

4.15. Provision of a rest area / rest breaks

4.16. Provision of time to attend medical appointments

4.17. Adjustment of the workplace environment

4.18. Modifications to the physical work environment

4.19. Permission not to wear a mask
